# Hippocampal network abnormalities explain amnesia after VGKCC-Ab related autoimmune limbic encephalitis

**DOI:** 10.1136/jnnp-2018-320168

**Published:** 2019-05-09

**Authors:** Clare Loane, Georgios P D Argyropoulos, Adriana Roca-Fernández, Carmen Lage, Fintan Sheerin, Samrah Ahmed, Giovanna Zamboni, Clare Mackay, Sarosh R Irani, Christopher R Butler

**Affiliations:** 1 Nuffield Department of Clinical Neurosciences, University of Oxford, Oxford, UK; 2 Institute of Cognitive Neuroscience, University College London Medical School, London, UK; 3 Unidad de Deterioro Cognitivo, Servicio de Neurología, Hospital Universitario Marques de Valdecilla, Santander, Spain; 4 Department of Neuroradiology, Oxford University Hospitals NHS Trust, Oxford, UK; 5 Oxford Centre for Human Brain Activity, University of Oxford, Oxford, UK

**Keywords:** encephalitis, memory, hippocampus, MRI

## Abstract

**Objective:**

Limbic encephalitis associated with antibodies to components of the voltage-gated potassium channel complex (VGKCC-Ab-LE) often leads to hippocampal atrophy and persistent memory impairment. Its long-term impact on regions beyond the hippocampus, and the relationship between brain damage and cognitive outcome, are poorly understood. We investigated the nature of structural and functional brain abnormalities following VGKCC-Ab-LE and its role in residual memory impairment.

**Method:**

A cross-sectional group study was conducted. Twenty-four VGKCC-Ab-LE patients (20 male, 4 female; mean (SD) age 63.86 (11.31) years) were recruited post-acutely along with age- and sex-matched healthy controls for neuropsychological assessment, structural MRI and resting-state functional MRI (rs-fMRI). Structural abnormalities were determined using volumetry and voxel-based morphometry; rs-fMRI data were analysed to investigate hippocampal functional connectivity (FC). Associations of memory performance with neuroimaging measures were examined.

**Results:**

Patients showed selective memory impairment. Structural analyses revealed focal hippocampal atrophy within the medial temporal lobes, correlative atrophy in the mediodorsal thalamus, and additional volume reduction in the posteromedial cortex. There was no association between regional volumes and memory performance. Instead, patients demonstrated reduced posteromedial cortico-hippocampal and inter-hippocampal FC, which correlated with memory scores (r = 0.553; r = 0.582, respectively). The latter declined as a function of time since the acute illness (r = -0.531).

**Conclusion:**

VGKCC-Ab-LE results in persistent isolated memory impairment. Patients have hippocampal atrophy with further reduced mediodorsal thalamic and posteromedial cortical volumes. Crucially, reduced FC of remaining hippocampal tissue correlates more closely with memory function than does regional atrophy.

## Introduction

Antibody-mediated limbic encephalitis (LE) is characterised by the subacute onset of amnesia and seizures and is commonly associated with antibodies against components of the voltage-gated potassium channel complex (VGKCC-Ab-LE): leucine-rich glioma-inactivated 1 (LGI1) and contactin-associated protein-like 2 (CASPR2).^1^ In the acute phase, MRI often reveals high T2 signal in the medial temporal lobes (MTL), a region crucial for memory processing.^2^ Although patients typically respond well to immunosuppressive therapy,^3^ some develop MTL atrophy and persistent cognitive impairment.^4–6^ In certain cohorts, 89% of patients develop hippocampal atrophy^7^ and 65% experience persistent memory deficits.^8^ The long-term cognitive outcome of VGKCC-Ab-LE has been investigated in a small number of group studies.^5 6 8^ These concur that memory impairment is the most salient feature, among other deficits.^6^

Nevertheless, the cognitive outcome in LE is difficult to predict and its relationship to brain damage poorly understood. Research so far has focused on the hippocampus, given that this is usually the locus of acute pathology, at least as detected on clinical MRI. Manual delineation of the hippocampus on MRI demonstrates atrophy following VGKCC-Ab-LE.^6 9^ However, clinical experience suggests that memory function correlates poorly with focal structural damage following VGKCC-Ab-LE; patients with apparently normal imaging may complain of marked memory impairment and vice versa.

It is less clear whether other areas within broader networks (eg, hippocampal-thalamic-neocortical^10 11^) are affected and contribute to residual memory impairment. Hippocampal damage in patients is also likely to disrupt functional connectivity (FC) with other regions supporting episodic memory within the partially overlapping limbic circuitry (eg, thalamus, posterior cingulate^12^) and the default-mode network.^13^ Such abnormalities have been reported in other encephalitides^14^ and neurodegenerative conditions.^15^ In a recent exploratory study in VGKCC-Ab-LE, FC alterations in large-scale networks were correlated with memory function, independent of hippocampal volume.^7^ Disrupted FC may thus be a better marker of amnesia in VGKCC-Ab-LE. Furthermore, understanding the impact of focal hippocampal damage on wider memory networks may inform understanding of cognitive deficits in other neurological diseases, as well as the neuroscience of human memory, since LE patients are often used as ‘lesion models’ to investigate hippocampal function.^16 17^

We thus investigated the neural correlates of long-term cognitive outcome in VGKCC-Ab-LE, in particular the possibility that memory function is determined to a greater extent by reduced hippocampal FC than by hippocampal atrophy. Using clinical assessment, cognitive evaluation, and structural and functional neuroimaging, we examined evidence of (1) extra-hippocampal structural damage, (2) reduced FC, and (3) a role of such disruptions in residual memory impairment following VGKCC-Ab-LE.

## Methods

### Subjects

Twenty-four patients were recruited into the University of Oxford’s Memory and Amnesia Project (MAP). Patients were identified from neurology clinics within UK NHS Trusts, presented with clinical features typical of LE,^2^ tested positive for serum VGKCC antibodies, reported persistent memory difficulties, and had no pre-existing neurological or psychiatric illness. All patients (20 male, 4 female) were in the post-acute phase (mean (SD) 5.22 (3.77) years) and came to Oxford for clinical assessment, neuropsychological testing, structural MRI, and resting state functional MRI (rs-fMRI).

Healthy controls were recruited (1) through local advertisement (n=39, of whom all underwent neuropsychology and 33 underwent structural and rs-fMRI) and (2) through the Oxford Project To Investigate Memory and Ageing (n=32; structural MRI only). Patients (mean (SD) age 63.86 (11.31) years) and controls (mean (SD) age 62.36 (12.09) years) were matched for age (t=−0.53, p=0.60), and were all fluent speakers of English.

### Clinical evaluation

Clinical assessment included evaluation of medical records for details of presentation onset, subsequent investigations, and response to treatment. Patients were invited for a research-oriented clinical appointment with a consultant neurologist (CRB) for detailed medical history and evaluation of ongoing clinical and cognitive problems.

### Antibody testing

Patient serum samples were tested for VGKCC antibodies from 125I-adendrotoxin labelled rabbit brain extract by routine immunoprecipitation.^2^ Cell-based assays for LGI1, CASPR2 and contactin-2 Abs used human embryonic kidney 293 cells transfected with DNAs encoding membrane-anchored LGI1, CASPR2 or contactin-2, which were incubated with sera.^1^

### Neuropsychological assessment

Neuropsychological tests were administered to assess premorbid intelligence, semantic memory and language, episodic memory, executive function, visuospatial/motor function and mood (online supplementary table S1). The p values were adjusted (‘p-corr’) using the Holm-Bonferroni sequential correction for multiple comparisons across all tests.

10.1136/jnnp-2018-320168.supp1 Supplementary data

An overall composite memory score was derived from memory tests in which patients showed impaired performance at group level as compared with controls (p-corr <0.05), by averaging the age-scaled, standardised scores across these tests. Composite subscores were also formed separately for verbal/visual recall/recognition memory.

### Acute clinical MRI ratings

A consultant neuroradiologist graded signal intensity, volume, and diffusion of left and right hippocampi in the acute T2-weighted clinical MRIs (online supplementary material S1). Abnormalities outside these structures were noted.

### Scanning procedures

We acquired 3D T1-weighted structural MRIs and resting-state Blood Oxygenation Level Dependent (BOLD)-weighted fMRI data (online supplementary material S2).

### Volumetry

**Figure 1:**
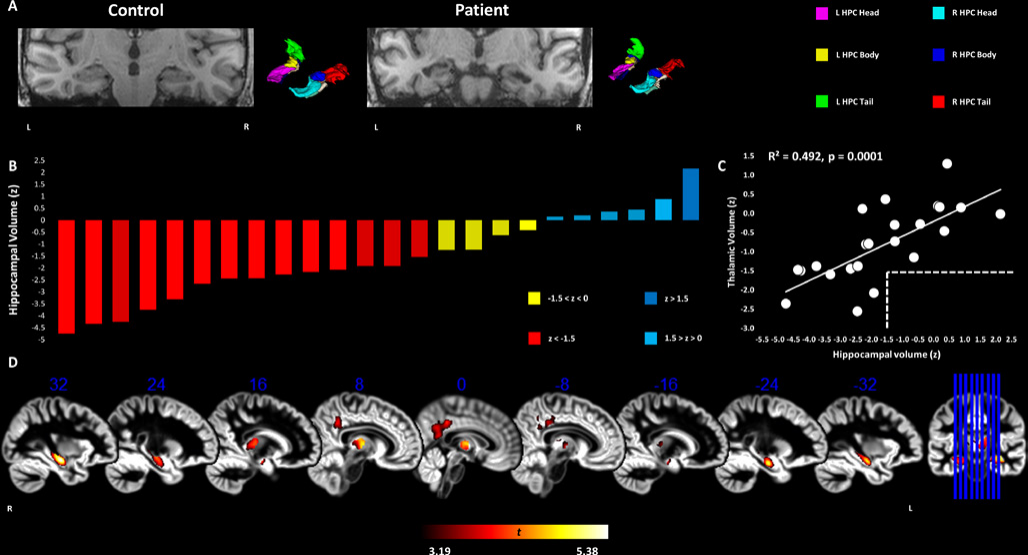
Grey matter volume reduction in the hippocampus, thalamus, and posteromedial cortex in VGKCC-Ab-LE patients. (A) T1-weighted MRI scans of an example control (left) and patient (right) with corresponding 3D rendering of manual segmentation masks. (B) Graph depicting total hippocampal volumes for patients (z-scores calculated on the basis of mean and SD of TIV-corrected volumes of individually age-matched controls). (C) Hippocampal and thalamic volumes correlated strongly across patients. Dashed lines indicate that there was no thalamic atrophy (z<−1.5) in the absence of hippocampal atrophy. (D) VBM maps overlaid on sagittal sections of DARTEL GM template in MNI space display bilaterally reduced GM volume in patients compared with controls (over and above age, sex, and TIV) in the hippocampus, thalamus, and precuneus-posterior cingulate. Clusters survive correction for non-stationary smoothness and FWE (p<0.05) across the whole brain for cluster size over p<0.001 (smoothing kernel: 8 mm FWHM). Heat bar indicates t-scores. FWE, family-wise error; FWHM, full width at half maximum; GM, grey matter; HPC, hippocampus; MNI, Montreal Neurological Institute; TIV, total intracranial volume; VBM, voxel-based morphometry; VGKCC-Ab-LΕ, limbic encephalitis associated with antibodies to components of the voltage-gated potassium channel complex.

Automated segmentation was conducted for the brainstem, thalamus, caudate nucleus, putamen and pallidum, using FSL FIRST (v. 6.0 http://www.fmrib.ox.ac.uk/fsl 18). For MTL structures (hippocampal head, body, tail; amygdala; perirhinal, entorhinal, parahippocampal cortices), since automated segmentation methods are less reliable in the presence of pathology, we used manual segmentation, which remains the gold standard for hippocampal volumetric measurements^19^ (24/24 patients; 46/65 controls) (figure 1A). Volumes were corrected for total intracranial volume (TIV) and expressed for each patient as z-scores based on the mean volumes and SDs of individually age-matched controls (online supplementary material S3).

In order to investigate the relationship between MTL/subcortical atrophy and memory impairment, atrophic volumes were entered in bivariate correlation analyses with composite memory scores. In order additionally to address the possibility that bilaterality of hippocampal atrophy, rather than overall volume loss, was associated with memory outcome, we trichotomised our patient group according to atrophy, using a traditional cut-off point of z<−1.5 SD below controls’ mean (eg, Gale and Hopkins^20^): the three subgroups comprised patients with bilateral (left and right hippocampal volumes z<−1.5 SD), unilateral (only left or right hippocampal volume z<−1.5 SD), or no atrophy (left and right hippocampal volumes z>−1.5 SD). We subsequently compared these subgroups on their composite memory scores.

### Voxel-based morphometry

To identify grey matter (GM) volume discrepancies between the control and patient groups at a whole-brain level, the T1-weighted images were analysed with VBM (online supplementary material S4), and the average volumes expressed in the clusters disclosed by the VBM contrasts were entered in bivariate correlation analyses with composite memory scores across patients. We also addressed the possibility that correlation of memory impairment with reduced volume may be a function of the spatial specificity of these clusters. Correlations were thus conducted separately at different levels of FWE (family-wise error)-correction (peak-/cluster-level) and image smoothing (4/8 mm FWHM (full width at half maximum)), and were corrected for multiple comparisons separately at each level.

### Functional connectivity analysis

Functional MRI data were preprocessed according to a default pipeline (online supplementary material S5) and were submitted to a series of FC analyses.

1. Multivariate pattern analysis: In order to identify seed regions for post-hoc seed-to-voxel FC analyses in an unbiased, data-driven fashion, we used multivariate pattern analysis (MVPA) (contrast: controls<>patients; covariates: age, sex; Online supplementary material S6).
2. Seed-to-voxel whole-brain FC analysis: We seeded from regions identified from the MVPA to explore FC between these regions and the rest of the brain. FC analyses (contrast: controls<>patients; covariates: age, sex) involved thresholding of FC maps at a voxel level of p<0.001 and FWE-corrected (p<0.05) at cluster- or peak-level.
3. Region of interest (ROI)-to-ROI FC analysis: In order to investigate the FC of regions identified in the MVPA with MTL structures with enhanced spatial precision, mean time series were extracted from the unsmoothed data in native space for all manually delineated MTL structures. We compared FC between controls and patients (covariates: age, sex), correcting at seed-level (p-corr) for multiple comparisons (online supplementary material S7).
4. Correlation with memory scores: To investigate whether reduced FC was associated with memory performance, correlation analyses between these FC measures and memory scores were conducted across patients and corrected for multiple testing (p-corr). FC measures were residualised against age, sex, and seed ROI volume in order to ensure that memory impairment was associated with genuine dysconnectivity (and not weaker FC due to fewer voxels in the ROI).

## Results

### Clinical

Key results on autoantibodies and acute MRI findings are presented in table 1 (see online supplementary table S2 for a summary). LGI1 was the prominent autoantibody, and the remaining patients did not differ in their acute and post-acute presentation from those that tested positive for the LGI1 autoantibody (online supplementary material S8). The patients with ongoing seizures did not differ on clinical, neuropsychological or radiological measures from those that had been seizure-free for over a year (online supplementary material S9). Likewise, no differences were noted between the patients that had presented acutely with faciobrachial dystonic seizures from the rest (online supplementary material S10). No correlations were identified among memory composite scores, acute T2 MRI ratings, antibody titre, or delay to treatment (all ps, p>0.05).

**Table 1:** Clinical details of patient group

Patient	Age	Sex	Antibody	Acute VGKCC Ab titre (pmol/L)	Onset to study inclusion (years)	Onset to immunosuppressive treatment (months)	Latest seizure to study inclusion (years)	Seizure type	Immunotherapy	Acute HPC T2 signal	Acute HPC volume	Acute HPC diffusion	Acute extra-HPC abnormalities	Follow-up HPC volume (z)
01	73.19	M	LGI1	4717	7	3	>4	GTCS; SPS	Oral steroids; PLEX; IVIG	R: normal L: very high	R: normal L: very enlarged	R: facilitated L: normal	L AMG: high T2 signal	R: −1.84 L: −2.68
02	76.04	F	VGKCC	801	6	1	<1	CPS; FBDS	Oral steroids; IVIG	R: normal L: high	R: mildly atrophic L: normal	R: facilitated L: facilitated	–	R: −1.40 L: −2.12
03	68.58	M	VGKCC	1936	6	12	>4	GTCS; CPS	Oral steroids; IVIG	R: normal L: very high	R: normal L: very enlarged	R: facilitated L: normal	L AMG, L ERC: high T2 signal	R: 1.75 L: −0.03
04	53.21	M	LGI1	336*	7	<1	>6	MCS	Oral steroids; IVIG	R: high L: normal	R: normal L: normal	R: normal L: facilitated	–	R: −4.67 L: −3.69
05	68.85	M	LGI1	1500	13	<1	>11	PCS; GTCS	Oral steroids; PLEX; IVIG	R: normal† L: normal†	R: normal† L: normal†	n/a†	–	R: −1.74 L: −2.27
06	64.44	M	LGI1	1735	6	8	>5	FBDS	Oral steroids; PLEX; IVIG	R: normal L: normal	R: normal L: normal	n/a	–	R: −0.58 L: −0.22
07	66.92	M	LGI1	416	11	5	>10	GTCS; CPS	PLEX; IVIG	R: normal L: high	R: normal L: enlarged	n/a	L/R AMG: high T2 signal	R: 1.79 L: 2.20
08	79.57	M	LGI1	4950	3	3	>1	GTCS; CPS	Oral steroids; PLEX; IVIG	R: high L: high	R: normal L: normal	R: normal L: facilitated	–	R: −0.59 L: 0.89
09	57.38	M	LGI1	3422	2	3	>1	CPS	Oral steroids; PLEX	R: very high L: high	R: enlarged L: enlarged	R: normal L: normal	–	R: −1.32 L: −1.06
10	57.39	M	LGI1	1306	9	38	<1	MCS	Oral steroids	n/a‡	n/a‡	n/a‡	–	R: −3.96 L: −5.30
11	46.70	M	LGI1	2249	1	3	<1	CPS	Oral steroids; IVIG	R: very high L: high	R: normal L: normal	n/a	–	R: −1.95 L: −2.17
12	55.05	M	LGI1/CASPR2	1228	9	2	>1	GTCS; CPS	Oral steroids; IVIG	R: high L: normal	R: atrophic L: normal	n/a	–	R: −2.58 L: −0.28
13	38.08	M	LGI1/CASPR2	378§	2	<1	>2	n/a	Oral steroids; IVIG	R: normal L: normal	R: mildly atrophic L: mildly atrophic	R: normal L: normal	–	R: −3.47 L: −2.97
14	82.46	M	LGI1	956	10	2	>9	n/a	Oral steroids; IVIG	R: high L: normal	R: mildly atrophic L: mildly atrophic	R: facilitated L: facilitated	–	R: −3.23 L: −2.06
15	55.18	M	LGI1	4091	3	12	>1	GTCS; CPS	Oral steroids	R: very high L: normal	R: normal L: normal	R: facilitated L: facilitated	–	R: −4.47 L: −2.71
16	56.03	M	LGI1/CASPR2	2878	4	7	>2	GTCS; CPS; MCS	Oral steroids; PLEX	R: very high L: normal	R: very enlarged L: normal	R: facilitated L: facilitated	–	R: −1.49 L: −3.31
17	56.93	M	VGKCC	1032	5	2	<1	CPS	Oral steroids; PLEX; IVIG	R: high L: normal	R: enlarged L: normal	n/a	–	R: −1.49 L: −2.27
18	76.59	M	LGI1	1430	2	3	<1	GTCS; CPS; MCS; FBDS	Oral steroids; IVIG	R: high L: high	R: enlarged L: enlarged	R: normal L: normal	L/R AMG: high T2 signal; enlarged	R: −0.03 L: 0.27
19	79.00	M	LGI1/CASPR2	1116	<1	3	<1	CPS	Oral steroids; IVIG	R: high L: high	R: mildly atrophic L: normal	R: normal L: normal	–	R: −0.04 L: 0.80
20	53.83	F	LGI1	1094	<1	2	<1	CPS	Oral steroids; IVIG	R: high L: high	R: normal L: normal	R: normal L: normal	–	R: −0.49 L: −0.72
21	75.07	F	VGKCC	n/a	1	n/a	>1	n/a	Oral steroids; IVIG	R: very high L: very high	R: enlarged L: enlarged	R: facilitated L: facilitated	–	R: −1.44 L: −2.70
22	65.32	M	LGI1	1400	2	2	>1	n/a	Oral steroids	R: high L: high	R: normal L: normal	R: normal L: normal	–	R: −1.30 L: −1.05
23	66.25	M	VGKCC	949	5	44	>5	n/a	IVIG	R: normal L: normal	R: normal L: normal	R: normal L: normal	–	R: 0.37 L: 0.28
24	60.49	M	VGKCC	n/a	6	n/a†‡§	>3	FBDS	Oral steroids; PLEX; IVIG	R: high L: high	R: atrophic L: atrophic	R: normal L: normal	L/R AMG: high T2 signal; atrophy	R: −4.49 L: −3.51

Acute titre per pmol/L.

No abnormality recorded.

*Reached 625 on repeat testing.

†Clinical letter mentions bilaterally high T2 signal in the hippocampi, disclosed by subsequent T2 clinical MRI (unavailable for rating).

‡Acute T2 clinical MRI unavailable for rating; clinical letter mentions bilaterally high T2 signal and enlargement in the hippocampi.

§Reached 1293 on repeat testing.

CASPR2, anti-contactin-associated protein-like 2; CPS, complex partial seizures; F, female; FBDS, faciobrachial dystonic seizures; GTCS, generalised tonic-clonic seizures; HPC, hippocampus; IVIG, intravenous immunoglobulin; LGI1, anti-leucine-rich glioma-inactivated 1; M, male; MCS, myoclonic seizures; PLEX, plasma exchange; SPS, simple partial seizures; VGKCC, anti-voltage-gated potassium channel complex; n/a, no information available; z, z-score calculated from mean and standard deviation of volumes of individually age-matched normal controls.

### Neuropsychological assessment

Patients were impaired on visual and verbal recall and verbal recognition memory measures; visual recognition memory was intact (overall memory composite score: controls: mean (SD) 0.74 (0.61); patients: mean (SD) −0.54 (0.83)). While patients scored higher than controls on the depression subscale of the Hospital Anxiety and Depression Scale, no participant scored in the severe range. Their performance on all other tests was no different from that of controls (table 2). Memory composite/subcomposite scores did not correlate with premorbid Full-Scale IQ (pFSIQ) or depression scores across patients (all rhos, rho|<0.28; p>0.21).

**Table 2:** Patients’ neuropsychological profile

Domain		Test	Subtest (measure)	Controls		Patients		Controls vs patients				
								Test	value	p-corr	Impaired	
				Median	IQR	Median	IQR				criterion	N
Episodic memory	Verbal recall	**WMS-III**	**Logical memory immediate recall (z**)	**0.33**	**1.75**	−**0.84**	**1.33**	**t**	**4.97**	**<** **0.001**	**≤−** **1.67**	**5**
			**Logical memory delayed recall (z**)	**0.67‡**	**1.75**	−**0.84**	**2.25**	**U**	**98.00**	**<** **0.001**	**≤−** **1.67**	**11**
			**Word list immediate recall (z**)	**0.67**	**2.00**	−**0.67**	**0.92**	**t**	**5.58**	**<** **0.001**	**≤−** **1.67**	**4**
			**Word list delayed recall (z**)	**1.33‡**	**1.00**	−**0.33‡**	**2.00**	**U**	**158.50**	**<** **0.001**	**≤−** **1.67**	**2**
		**D&P**	**People (z**)	−**0.33**	**1.67**	−**1.33**	**1.09**	**t**	**3.49**	**0.026**	**≤−** **1.67**	**7**
	Visual recall	**ROCFT**	**Immediate recall (z**)	**1.32‡**	**1.82**	**0.00**	**2.97**	**U**	**215.50**	**0.018**	**≤−** **1.67**	**7**
			**Delayed recall (z**)	**1.38‡**	**1.87**	**0.00 ‡**	**3.42**	**U**	**220.50**	**0.034**	**≤−** **1.67**	**8**
		D&P	Shapes (z)	0.67	1.00	0.00	1.75	Wt	3.09	0.088	**≤−** **1.67**	10
	Verbal recognition	**WMS-III**	**Word list recognition (z**)	**0.67‡**	**1.00**	**0.00 ‡**	**1.58**	**U**	**193.50**	**0.003**	**≤−** **1.67**	**4**
		**RMT**	**Words (z**)	**1.00‡**	**1.67**	**0.33 ‡**	**1.67**	**U**	**241.50**	**0.046**	**≤−** **1.67**	**3**
		**D&P**	**Names (z**)	**0.33**	**2.00**	−**0.67**	**1.66**	**t**	**3.21**	**0.046**	**≤−** **1.67**	**4**
	Visual recognition	RMT	Scenes (z)	1.00	1.01	0.59**‡**	1.96	U	247.00	0.572	**≤−** **1.67**	1
		D&P	Doors (z)	0.67	1.33	−0.17	1.75	t	1.89	0.768	**≤−** **1.67**	7
		RMT	Faces (z)	−0.33	2.66	−0.33	1.66	t	0.61	>0.999	**≤−** **1.67**	4
Executive function		WMS-III	Digit span (z)	1.00	1.00	0.17	1.59	t	2.89	0.108	**≤−** **1.67**	1
		DKEFS Fluency	Letter fluency (z)	1.33	1.17	0.00	1.66	t	2.64	0.203	**≤−** **1.67**	1
			Letter vs category fluency (z)	0.33	1.33	1.00	1.00	t	−2.13	0.564	**≤−** **1.67**	0
			Category switching vs fluency (z)	0.00	1.67	0.33	1.00	t	−1.83	0.768	**≤−** **1.67**	0
		DKEFS Trails	Number and letter sequencing (z)	1.00**‡**	0.66	0.00	1.41	U	242.50	0.088	**≤−** **1.67**	0
			Letter-number switching vs number and letter sequencing (z)	−0.33	0.67	−0.17	1.42	Wt	−0.39	>0.999	**≤−** **1.67**	1
Intelligence, semantic memory, and language		WASI/WASI-II	Vocabulary (z)	1.50	1.30	0.80	1.30	t	2.55	0.234	**≤−** **1.67**	0
		WASI/WASI-II	Similarities (z)	0.95**‡**	0.80	0.85	1.55	U	238.50	0.768	**≤−** **1.67**	0
		NART	p-FSIQ (z)	1.44**‡**	0.68	1.04**‡**	0.71	U	260.00	0.245	**≤−** **1.67**	0
		GNT (z)		0.88 **‡**	0.98	0.15	1.91	U	295.50	0.693	**≤−** **1.67**	3
		C&CT (z)		0.34	1.30	0.02**‡**	1.30	U	324.50	0.831	**≤−** **1.67**	4
Mood and anxiety		**HADS**	**Depression (raw score; max=21**)	**1.00‡**	**1.00**	**3.00‡**	**4.50**	**U**	**182.00**	**0.008**	≥15 *	**0**
			Anxiety (raw score; max=21)	3.50**‡**	4.00	5.00	7.00	U	253.00	0.311	≥15 *	1
Visuospatial and motor function		DKEFS Trails	Visual scanning (z)	0.67**‡**	1.67	0.00	1.34	U	359.50	>0.999	**≤−** **1.67**	3
			Motor speed (z)	0.67**‡**	1.00	0.33**‡**	0.67	U	367.50	>0.999	**≤−** **1.67**	3
		VOSP	Cube analysis (raw score; max=10)	10.00**‡**	1.00	10.00**‡**	1.00	U	354.00	>0.999	≤6†	2
			Dot counting (raw score; max=10)	10.00**‡**	0.00	10.00**‡**	0.00	U	378.50	>0.999	≤8†	0
			Position discrimination (raw score; max=20)	20.00**‡**	0.00	20.00**‡**	1.00	U	387.00	>0.999	≤18†	4
		ROCFT	Copy (ranked percentile ranges)	> 16^th^ percentile**‡**	–	> 16th percentile**‡**	–	U	421.50	>0.999	**≤16th percentile**	2

Controls (n = 39; 26M:13F; age: mean (SD) 60.86 (11.61) years) and patients (n = 24; 20M:4F; age: mean (SD) 63.45 (11.27) years) did not differ in age at assessment (t =−0.87, p=0.39) or M:F ratio (χ^2^ = 2.10, p=0.15).

*Cut-off score for severe range.

†5% cut-off score; ‘impaired’: number of patients below the cut-off score per test.

‡Shapiro-Wilk test: p<0.05.

Bold, p-corr <0.05; C&CT, Camel and Cactus Test; DKEFS, Dellis-Kaplan Executive Function System; D&P, Doors and People Test; GNT, Graded Naming Test; HADS, Hospital Anxiety and Depression Scale; NART, National Adult Reading Test; RMT, Recognition Memory Test; ROCFT, Rey-Osterrieth Complex Figure Test; U, Mann-Whitney U; VOSP, Visual Object and Space Perception Battery; WASI/WASI-II, Wechsler Abbreviated Scale of Intelligence; WMS-III, Wechsler Memory Scale III; Wt, Welch’s t-test; pFSIQ, premorbid Full-Scale IQ; p-corr, p values adjusted for Holm-Bonferroni sequential correction for multiple comparisons across all tests; t, Student’s t-test; z, age-scaled standardised scores.

### Volumetry

Patients showed bilateral hippocampal atrophy and reduced thalamic volumes. The rest of their volumes did not differ from control volumes (table 3). Hippocampal and thalamic volumes did not correlate with pFSIQ or depression (Hospital Anxiety and Depression Scale (HADS)) across patients (all rhos, rho|≤0.305; all ps, p≥0.167).

**Table 3:** Volumetry of medial temporal lobe and subcortical structures

Structure		Hemisphere	Mean (z)	SD (z)	vs 0		n atrophic	Correlation with memory composite score	
					t	p-corr			
								R	p-corr
HPC	**Head**	**R**	−**1.71**	**1.79**	−**4.68**	**0.003**	14	−0.035	>0.999
		**L**	−**1.71**	**1.52**	−**5.53**	**0.0003**	16	0.181	>0.999
	**Body**	**R**	−**0.95**	**1.07**	−**4.37**	**0.006**	7	0.043	>0.999
		L	−0.81	1.47	−2.69	0.25	8	n/a	
	Tail	R	−0.67	1.56	−2.11	0.83	9		
		L	−0.58	1.51	−1.87	>0.999	7		
ERC		R	−0.70	1.08	−3.18	0.09	3		
		L	−0.77	1.35	−2.81	0.21	8		
PRC		R	−0.43	1.25	−1.66	>0.999	1		
		L	−0.21	1.13	−0.90	>0.999	2		
PHC		R	−0.50	0.89	−2.76	0.23	2		
		L	−0.36	1.20	−1.46	>0.999	5		
AMG		R	0.06	1.36	0.21	>0.999	3		
		L	−0.14	1.72	−0.39	>0.999	4		
TPC		R	0.20	1.13	0.87	>0.999	1		
		L	0.30	1.01	1.46	>0.999	0		
**Thalamus**		**R**	−**0.75**	**0.95**	−**3.86**	**0.02**	5	0.187	>0.999
		**L**	−**0.89**	**1.04**	−**4.20**	**0.008**	6	0.173	>0.999
Caudate nucleus		R	−0.03	1.00	−0.14	>0.999	1	n/a	
		L	−0.02	1.36	−0.06	>0.999	2		
Nucleus accumbens		R	−0.16	0.87	−0.88	>0.999	1		
		L	−0.40	1.30	−1.51	>0.999	4		
Pallidum		R	−0.28	1.10	−1.25	>0.999	3		
		L	−0.22	1.32	−0.81	>0.999	3		
Putamen		R	−0.23	1.10	−1.01	>0.999	3		
		L	−0.38	0.94	−2.00	0.98	3		
Brainstem			−0.33	1.08	−1.49	>0.999	3		

For controls and patients, MTL structures (hippocampal head, body, tail, amygdala, entorhinal, parahippocampal, perirhinal, temporopolar cortex) were manually segmented, and other subcortical structures (thalamus, pallidum, putamen, brainstem, nucleus accumbens, and caudate nucleus) were automatically segmented (FSL FIRST). All volumes were TIV-corrected and expressed as z-scores, based on the mean and SD of patients’ individually age-matched controls (± 10 years of age). Comparisons are hence conducted as one-sample t-tests versus 0 (control mean); p-corr: two-tailed, adjusted with Holm-Bonferroni sequential correction for multiple testing. Bold: p-corr <0.05; r: Pearson’s correlation coefficient; of the volumes that showed reduction in patients relative to controls, none showed correlation with composite memory scores. No correlation was found with total/left/right hippocampal atrophy at uncorrected levels (all ps, p>0.88); ‘n atrophic’: number of patients whose volumes fall below −1.5 SD from those of their age-matched healthy controls.

AMG, amygdala; ERC, entorhinal cortex; HPC, hippocampus; MTL, medial temporal lobe; PHC, parahippocampal cortex; PRC, perirhinal cortex; TIV, total intracranial volume; TPC, temporopolar cortex.

Total hippocampal volumes fell below 1.5 SDs from controls for 14/24 patients (figure 1B). Hippocampal and thalamic volume reduction were positively correlated. There was no thalamic atrophy (z<−1.5) in the absence of hippocampal atrophy (0/10 patients), whereas 9/14 patients with hippocampal atrophy showed non-atrophic thalamic volumes (z>−1.5), and all 5/5 patients with thalamic atrophy showed hippocampal atrophy (figure 1C).

#### Relationships with memory scores

However, composite memory scores did not correlate with patients’ hippocampal or thalamic volumes (table 3; S3), and did not differ among patients with bilateral (n=9), unilateral (n=5) or no hippocampal atrophy (n=10) (Kruskal-Wallis test: H=0.004, p>0.99).

### Whole-brain voxel-based morphometry

Patients showed bilateral volume reduction in the hippocampal heads and bodies, the mediodorsal thalamus, and the posterior cingulate-precuneus (figure 1D; table 4). Patients’ pFSIQ and depression (HADS) scores did not correlate with these volumes (all rhos, rho|≤0.322; all ps, p≥0.143). Hippocampal and thalamic volumes were strongly correlated.

**Table 4:** Whole-brain voxel-based morphometry (grey matter volume)

Smoothing (FWHM)	FWE-correction level	Peak				Cluster		Correlations		
								Memory scores		GM volumes
		t	x (mm)	y (mm)	z (mm)	kE (nvox)	Structure	r	p-corr	
4 mm	Peak	5.90	−25	−16	−22	197	L HPC head	0.37	0.225	L HPC head – R body: r=0.61, p-corr=0.002 L – R HPC head: r=0.64, p-corr=0.002 R HPC head – body: r=0.82, p-corr<0.0001
		5.75	37	−27	−11	36	R HPC body	0.10	0.986	
		5.57	31	−13	−17	61	R HPC head	0.15	0.986	
	Cluster size	5.90	−25	−16	−22	2156	L HPC head/body	0.32	0.424	R-L HPC: r=0.64, p-corr=0.005 R HPC – R Thal: r=0.54, p-corr=0.035 Rest of p-corr>0.15
		5.75	37	−27	−11	2721	R HPC head/body	0.03	>0.999	
		5.22	19	−28	5	3085	R Thal	0.34	0.424	
		4.74	-2	−39	40	2086	L/R PCC/PrCu	−0.02	>0.999	
8 mm	Peak	5.38	32	−15	−16	592	R HPC head/body	0.35	0.267	R-L HPC: r=0.65, p-corr=0.0006 R HPC – R Thal: r=0.55, p-corr=0.006 L HPC – R Thal: r=0.46, p-corr=0.02
		5.19	−27	−15	−20	200	L HPC head/body	0.11	0.599	
		4.91	6	−12	7	179	R MD Thal	0.36	0.267	
	Cluster size	5.38	32	−15	−16	12 710	L/R HPC/Thal	0.31	0.294	r=0.32, p-corr=0.13
		4.16	-1	−38	38	6606	L/R PCC/PrCu	−0.03	0.900	

Contrast: controls > patients; nuisance covariates: age, sex, TIV; voxel dimensions: 1 mm^3^; individual voxel threshold: p<0.001. The average GM volume from each cluster was extracted from each participant, residualised against age, sex, and TIV, and entered in a bivariate correlation analysis with the memory composite score. No correlation was noted with the GM volume of any cluster; hippocampal volume reduction strongly correlated with thalamic volume reduction in patients; p-corr: p values of the bivariate correlations conducted are adjusted for multiple testing using the Holm-Bonferroni sequential correction method, separately for the number of clusters disclosed in each of the four separate VBM analyses conducted.

GM, grey matter; HPC, hippocampus; L/R, left/right; MD, mediodorsal; PCC, posterior cingulate cortex; PrCu, precuneus; TIV, total intracranial volume; Thal, thalamus; VBM, voxel-based morphometry.

#### Relationships with memory scores

Nevertheless, volumes did not correlate with the overall composite memory scores, irrespective of smoothing or FWE-correction levels (table 4). Correlations were only found at uncorrected levels between verbal recall memory scores and GM volume of the left hippocampal clusters (all rs, 0.42<r < 0.45; all ps, 0.025<p<0.040; Online supplementary table S4).

### Functional connectivity analysis

**Figure 2:**
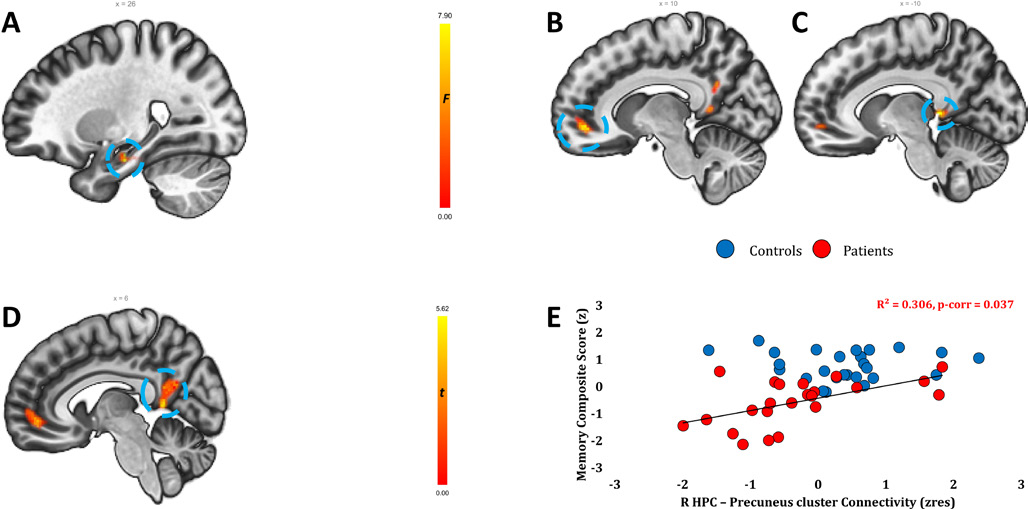
Resting-state functional connectivity (FC) analyses (whole-brain). (A) A whole-brain, voxel-to-voxel resting-state FC analysis (MVPA) contrasting patients and controls demonstrated that the two groups differed in the FC of the right hippocampus with the rest of the brain (cluster-level p-FWE=0.018; kE=128 vox; peak: 26, –16, −22). Heat bar indicates F values. (B–D) Regions showing reduced FC with patients’ right hippocampi (F contrast: controls <>patients; nuisance covariates: age, sex). (B) Medial prefrontal/paracingulate cortex (cluster-level p-FWE<0.001; kE=547 vox; peak: 10, 48, –6). (C) Left posterior cingulate cluster, extending to precuneus, thalamus, and hippocampal tail (cluster-level p-FWE=0.024, kE=109 vox; peak: –10, –40, 4). (D) Right precuneus-posterior cingulate cluster (cluster-level p-FWE<0.001, kE=434 vox; peak: 6, –48, 6); heat bar indicates T values. (A–D) Clusters are displayed on sagittal sections of ICBM template in MNI space. (E) Correlation between right hippocampal-precuneal (C) FC (residualised for age, sex, and right hippocampal seed volume) with (age-scaled standardised) composite memory scores in patients, surviving correction for multiple testing for all correlations (rest of p-corr>0.459). FWE, family-wise error; HPC, hippocampus; MVPA, multivariate pattern analysis.

**Figure 3:**
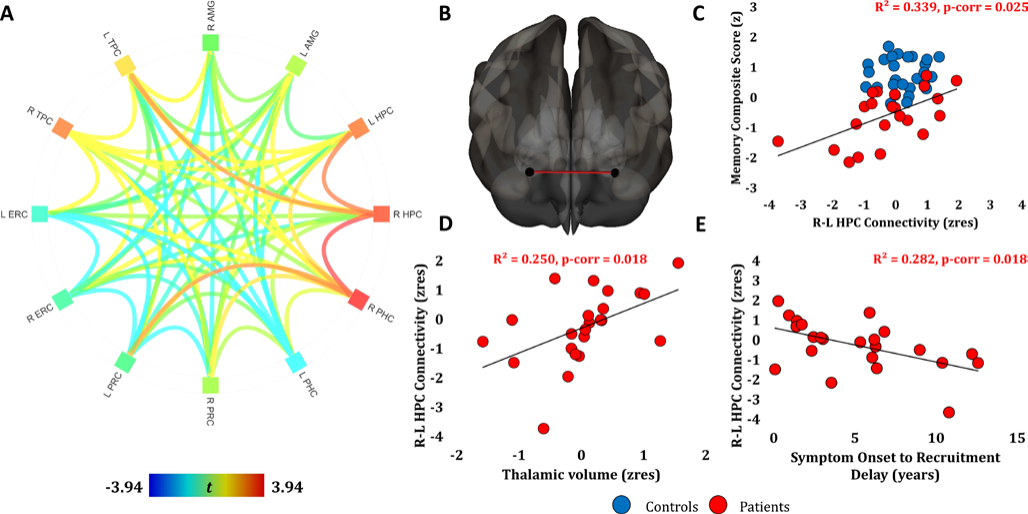
Resting-state functional connectivity (FC) analyses (MTL). (A) ROI-to-ROI FC analysis for MTL structures (BOLD time-series extracted from unsmoothed data in native space; contrast: patients <>controls; nuisance covariates: age, sex); orange and red lines indicate FC values that survive Holm-Bonferroni correction (p-corr<0.05) for multiple comparisons (R HPC – L HPC; R HPC – R PHC; R HPC – L TPC; R PHC – L PRC). Colour in squares: average FC of each ROI with other ROIs. (B, C) Inter-HPC FC correlated with (age-scaled standardised) memory composite scores in patients (residualised against age, sex, and total HPC seed volume), and survived correction for multiple testing for all correlations of FC measures with memory scores (rest of p-corr>0.459). (D) Inter-HPC FC correlated with thalamic volumes in patients (residualised against age, sex, and total HPC volume). (E) Patients’ inter-HPC FC (residualised for age, sex, and total HPC volume) declined as a function of time since symptom onset. (D, E) Correlations survived correction for multiple testing for the two FC measures that correlated with memory scores. AMG, amygdala; BOLD, blood oxygenation level dependent; ERC, entorhinal cortex; HPC, hippocampus; L/R: left/right hemisphere; MTL, medial temporal lobe; PHC, parahippocampal cortex; PRC, perirhinal cortex; ROI, region of interest; TPC, temporopolar cortex.

We subsequently examined FC abnormalities across the whole brain (MVPA). Patients differed from controls in the FC of the right hippocampus (figure 2A). In order to identify the regions with which the right hippocampus showed abnormal FC, as well as to avoid the possibility that the right hippocampal cluster disclosed above resulted from suboptimal spatial coregistration of patients’ functional data to their atrophic hippocampi, we conducted a whole-brain seed-to-voxel FC analysis, seeding from the right hippocampus in native space (unsmoothed time series). Patients showed reduced FC with the medial prefrontal and posteromedial cortices (posterior cingulate-retrosplenial cortex-precuneus), extending to the left hippocampal tail (figure 2B–D). We further investigated reduced FC within the MTL to enhance spatial specificity (ROIs in native space, unsmoothed time series). Patients showed reduced FC between the right hippocampus and the left hippocampus, the right parahippocampal cortex and the left temporopolar cortex, and also between the right parahippocampal and left perirhinal cortices (figure 3A).

#### Relationships with memory scores

Patients’ weaker inter-hippocampal FC correlated with their lower overall composite memory scores (r=0.582, p-corr=0.025), with all three composite subscores showing this correlation (visual recall: r=0.444, p=0.039; verbal recall: r=0.482, p=0.020; verbal recognition: r=0.574, p=0.004). Posteromedial cortico-hippocampal FC also correlated with patients’ memory scores (overall: r=0.553, p-corr=0.037; visual recall: r=0.547, p=0.008; verbal recall: r=0.444, p=0.034; verbal recognition: r=0.477, p=0.021) (figures 2E and 3C). Inter-hippocampal and posteromedial cortico-hippocampal FC did not correlate with pFSIQ or depression scores (HADS) across patients (all rhos, rho|<0.010; all ps, p>0.700), and did not differ among patients with bilateral, unilateral, or no hippocampal atrophy (both Hs, H<3.300; both ps, p>0.190). Posteromedial cortico-hippocampal FC did not correlate with volume reduction in the same region (r=−0.20, p=0.36). Inter-hippocampal FC declined as a function of thalamic atrophy over and above hippocampal atrophy (r=0.500, p-corr=0.018), and as a function of the delay between symptom onset and recruitment to the study (r=-0.531, p-corr=0.018; figure 3D,E). In a series of exploratory multiple stepwise linear regression analyses (independent variables: structural, functional abnormalities), reduced hippocampal FC measures were found to be the only predictors of memory impairment (online supplementary table S5).

## Discussion

We conducted a detailed investigation of neuropsychological performance and structural and functional brain imaging following VGKCC-Ab-LE, in order to identify: (1) extra-hippocampal structural damage; (2) reduced hippocampal FC; (3) the role of those in residual cognitive dysfunction following VGKCC-Ab-LE.

We identified bilateral hippocampal atrophy that was highly focal within the MTL, along with correlated volume reduction in the mediodorsal thalamus and precuneus-posterior cingulate, which are connected with the hippocampus within the limbic network.^12 21^ Our cohort showed disrupted hippocampal FC within the MTL as well as with anterior and posterior medial cortical regions. Inter-hippocampal FC declined as a function of time from the acute stage of the illness and as a function of thalamic volume reduction above and beyond hippocampal atrophy. Neuropsychological testing revealed a pure amnesic syndrome. Importantly, it was the FC of residual hippocampal tissue, rather than volume, which correlated with patients’ memory performance.

### Acute stage clinical features

Τhe predominant antigenic target was LGI1. The percentage of patients with hippocampal high T2 signal (83%) was in line with previous reports (71%^22^; 73%^6^; 78%^3^). The neuropathology underlying acute hippocampal signal change in VGKCC-Ab-LE is unknown. Interestingly, the acute diffusion profile in VGKCC-Ab-LE starkly contrasted with that of clinically similar conditions (eg, herpes simplex encephalitis), where restricted, as opposed to facilitated, diffusion within the hippocampal formation^23^ is more commonly observed. This may relate to differences in the types of cerebral oedema, that is, vasogenic in VGKCC-Ab-LE versus cytotoxic oedema elsewhere.

### VGKCC-Ab-LE is associated with focal hippocampal damage and dysfunction

Capitalising on the strengths of manually segmented MTL structures and whole-brain VBM, we found bilateral (especially anterior) hippocampal atrophy that was highly focal within the MTL. Our findings are consistent with the well-recognised focal T2 signal abnormalities in the hippocampus in the acute phase,^24^ and extend prior studies^6 9^ by documenting normal volumes of extra-hippocampal MTL and other subcortical structures. Since the VGKCC is found throughout the peripheral and central nervous systems,^1^ the reason for such anatomical specificity in VGKCC-Ab-LE pathology is unclear. High LGI1 density within the hippocampus^25^ or local blood-brain barrier vulnerability may play a role.^26^ Nevertheless, the relatively focal nature of this disease supports its use as a lesion model for exploring the cognitive role of the hippocampus.^27^

### VGKCC-Ab-LE-related hippocampal atrophy is associated with thalamic volume reduction

The finding of bilateral volume reduction in the thalamus and the precuneus-posterior cingulate cortex in VGKCC-Ab-LE is novel and deserves further investigation. Several facts point towards the interpretation of such reduction as resulting from Wallerian degeneration through an extended limbic circuit^12 21^: (1) no thalamic or neocortical abnormality was observed in the acute clinical MRI; (2) volume reduction in the thalamus was strongly correlated with that in the hippocampus, and there was no thalamic atrophy in the absence of hippocampal atrophy; (3) animal models provide mechanistic reasons why the focus of acute pathology in LGI1 LE patients might be constrained to the MTL,^28^ suggesting that changes elsewhere may be consequent to MTL damage, rather than to antibodies per se; (4) thalamic abnormalities have been observed in other studies of human hippocampal damage— volume reduction in the thalamus has been noted in developmental amnesia, where hippocampal atrophy is held to occur focally within the MTL,^29^ and also in the limbic network following fornix damage^30^; (5) the concomitant volume reduction in the posteromedial cortex dovetails with the idea of network-specific degeneration across the hippocampal-diencephalic-cingulate circuitry,^21^ rather than with more widespread, autoantibody-driven pathology.

### VGKCC-Ab-LE causes focal amnesia with spared visual recognition memory

Our patients demonstrated amnesia with no deficits in other domains, in contrast to other reports (eg, Heine *et al* 7). This is consistent with findings that VGKCC-Ab-LE patients often make substantial recovery post-immunotherapy in executive function, yet present with residual amnesia.^5^ The focal nature of our patients’ impairment is of importance in the interpretation of their brain abnormalities.

Patients showed striking preservation of visual recognition memory despite profound impairment of verbal and non-verbal recall and verbal recognition memory. There is a long-standing debate about whether recall and recognition memory rely on dissociable neurocognitive processes.^30 31^ One view is that associative processes critical for recollection are dependent on the hippocampus, whereas familiarity is driven by non-hippocampal regions, particularly the perirhinal cortex.^32^ Accordingly, hippocampal atrophy should impair recollection but spare familiarity and (partly) recognition memory.^33^ Our finding that visual recognition memory is spared in patients with focal hippocampal damage and intact perirhinal cortex partly supports this view. However, we found impairment in all tests of verbal recognition. This is consistent with the idea that spared familiarity processes play a greater role in recognition memory for novel visual stimuli (eg, faces, doors), than they do for previously encountered, semantically laden stimuli (eg, words).^34^ Finer-grained tasks are necessary to explore these findings in detail and further characterise the behavioural phenotype of autoimmune LE.

### Network dysfunction rather than focal atrophy predicts memory performance

Ever since the first description of patient H.M. 60 years ago,^35^ it has been recognised that hippocampal damage, as occurs in conditions such as Alzheimer’s disease, temporal lobe epilepsy and LE, may result in focal amnesia. However, little is known about reduced FC that may be triggered by late-onset hippocampal damage, and its impact on memory performance.

Indeed, we found that it was the FC, and not the volume of the hippocampus, that predicted patients’ memory outcome. Our findings do not contradict the well-established role of the hippocampus in memory. Instead, they highlight the significance of an underappreciated aspect of hippocampal damage in explaining hippocampal amnesia, namely, the abnormal FC of residual hippocampal tissue with broader memory networks. Arguably, haemodynamic properties of residual hippocampal tissue may reflect more accurately the extent to which the damaged hippocampus can interact with broader networks in supporting memory processes than hippocampal volumes derived from structural MRI.

In particular, memory impairment was associated with reduced posteromedial cortico-hippocampal and inter-hippocampal FC. The latter declined as a function of time from symptom onset. Inter-hippocampal FC has been linked to memory performance in young adults^36^ and patients with traumatic brain injury.^37^ It has also been reported to decline from healthy controls to mild cognitive impairment and even more so in Alzheimer’s disease,^38^ and may reflect the integrity of the dorsal hippocampal commissure.^39^ The fact that patients’ reduced (primarily mediodorsal) thalamic volumes positively correlated with this FC dovetails with evidence supporting a complex interplay between midline thalamic nuclei and the hippocampus in mnemonic processes.^40^ The relationship of amnesia with reduced posteromedial cortico-hippocampal FC is consistent with the well-established role of these cortical regions within the default-mode network.^13^ This network supports episodic memory processes, and its disruption is well-documented in episodic memory impairment (eg, Finke *et al*, Buckner *et al*,^14 15^).

### Limitations and future directions

We acknowledge a number of limitations and future directions that should be taken. Sample size was limited by the rarity of the condition, and larger cohorts may reveal more subtle brain-behaviour relationships. Furthermore, our study did not include disease controls, so we are unable to comment on whether our findings generalise to other types of LE. The 3T field strength prevented us from identifying subfield-specific atrophy, as reported elsewhere.^9^ Moreover, we observed no relationship among acute hippocampal abnormalities, delay to treatment, time since the acute stage of the illness, acute-stage antibody titres, and post-treatment memory outcome. Previous studies that have examined indicators of cognitive prognosis in VGKCC-Ab-LE show mixed results (Irani *et al*, Finke *et al* 1 6 vs Malter *et al*, Miller *et al* 8 9). There are many possible reasons for this inconsistency (variability in clinical presentation, types of immunotherapy, cognitive measures employed, statistical power). This question should be answered by large, prospective, multicentre studies. Finally, our cross-sectional approach prevented us from examining the time-course of extra-MTL abnormalities. The question of whether Wallerian degeneration underlies neuronal loss through wider memory networks following hippocampal damage should be addressed with longitudinal analyses.
