# Supplementary material for: Hippocampal network abnormalities explain amnesia after VGKCC-Ab related autoimmune limbic encephalitis
Source: J Neurol Neurosurg Psychiatry. 2019 May 9;90(9):965–74. doi: 10.1136/jnnp-2018-320168 (PMC6820158; doi:10.1136/jnnp-2018-320168)
Supplement: Supplementary data [file jnnp-2018-320168supp001.pdf]

Online only supplementary material

Supplementary Table 1: Neuropsychological Tests

Domain	Test
General intelligence, semantic memory, and language	National Adult Reading Test (NART) [1]
	Camel and Cactus Test (C&CT) [2]
	Graded Naming Test (GNT) [3]
	Weschler Abbreviate Scale of Intelligence (WASI/WASI-II) Vocabulary and Similarities [4]
executive function	Wechsler Memory Scale III (WMS-III) digit span [5]
	Delis-Kaplan Executive Function System (DKEFS) [6] Trail Making Test and Verbal Fluency
visuospatial and motor function	Rey-Osterrieth Complex Figure Test (ROCFT) [7] copy
	DKEFS visual scanning; motor speed [6]
	Visual Object and Space Perception (VOSP) cube analysis; dot counting; position discrimination [8]
episodic memory	WMS-III (immediate and delayed verbal recall: Logical Memory I; II; Word List I; II; recognition) [5]
	ROCFT [7] (immediate; delayed visual recall)
	Warrington Recognition Memory Test (faces; words) (RMT) [9]
	Warrington Topographical Memory test (scenes) (RMT) [10]
	Doors and People test (D&P) Names: verbal recognition; People: verbal recall; Doors: visual recognition; Shapes: visual recall [11]
Depression and anxiety	Hospital Anxiety and Depression Scale (HADS) [12]

Loane C, *et al. J Neurol Neurosurg Psychiatry* 2019; 0:965–974. doi: 10.1136/jnnp-2018-320168

S1. Acute clinical MRI ratings

Acute clinical scans were available for 23/24 patients. T2 signal intensity and volume were evaluated in these MRIs. Diffusion was evaluated in 17 patients. The consultant neuroradiologist (FS) graded T2 signal intensity (1 = normal, 2 = high, 3 = very high), volume (1 = very atrophic, 2 = mildly atrophic, 3 = normal, 4 = enlarged, 5 = very enlarged) and diffusion (1 = normal, 2 = facilitated, 3 = restricted) of both left and right hippocampi.

Loane C, *et al. J Neurol Neurosurg Psychiatry* 2019; 0:965–974. doi: 10.1136/jnnp-2018-320168

S2. Research Scan Procedures

Image acquisition for research scans was conducted on a Siemens 3T Trio system using a 32-channel head coil (University of Oxford Centre for Clinical Magnetic Resonance Research). We acquired 3D T1-weighted structural MRIs (MPRAGE sequence; echo time = 4.7ms, repetition time = 2040ms, 8° flip angle, field of view = 192mm, voxel size = 1 x 1 x 1mm) and resting-state BOLD-weighted fMRI data (Gradient Echo EPI sequence; 180 volumes; slice thickness = 3.5mm, echo time = 28ms, repetition time = 2410ms, 89° flip angle, field of view = 192mm, voxel size = 3 x 3 x 3.5mm). Subjects were instructed to lay still, watch a fixation cross on the in-scanner projector and not to fall asleep.

S3. Volumetry

Manual delineation of MTL structures was conducted by a trained researcher (ARF) using ITK-SNAP [13] (protocol: https://www.ndcn.ox.ac.uk/files/research/segmentation_protocol_medial_temporal_lobes.pdf). Segmentation procedures were based on established and published atlases and protocols [14,15]. Intra-rater reliability was measured using intra-class coefficient correlations on a random selection of 24 hemispheres (12 patients; 12 controls) segmented twice by ARF with an interval of at least 4 months between segmentations. The intra-rater reliability results were at least as good those reported in the literature for manual segmentations of the MTL [16] for the hippocampus (0.982), amygdala (0.803), and the temporopolar (0.96), perirhinal (0.904), entorhinal (0.816), and parahippocampal cortices (0.93). Automated segmentation was used for the brainstem, thalamus, caudate nucleus, putamen, and pallidum. Volumes were segmented in each hemisphere and corrected for total intra-cranial volume (TIV) derived from the unified segmentation procedure [17] in SPM12. For each patient, volumes were expressed as z scores based on the mean volumes and SDs of individually age-matched (+/- 10 years of age) controls (number of controls per patient: mean = 22; SD = 7).

S4. VBM

To identify GM volume discrepancies between groups at a whole-brain level, the T1-weighted images were analyzed with VBM ([18]) using the Statistical Parametric Mapping software (SPM12 v7219; http://www.fil.ion.ucl.ac.uk/spm/software/spm12) running in Matlab R2017b. Images were examined for scanner artefacts, reoriented to have the same point of origin (anterior commissure) and spatial orientation, bias-corrected to remove intensity non-uniformities, and segmented into GM, white matter (WM), and cerebrospinal fluid (CSF), using the unified segmentation procedure [17]. The diffeomorphic anatomical registration through the exponentiated lie algebra (DARTEL) toolbox was applied to all participants' GM, WM, and CSF, to refine inter-subject registration, and study-specific GM templates were generated [19]. After affine registration of the GM DARTEL templates to the tissue probability maps in MNI (Montreal Neurological Institute, Quebec, Canada) space, non-linear warping of GM images was performed to the DARTEL GM template in MNI space. Voxel values in the tissue maps were modulated by the Jacobian determinant (calculated during spatial normalization), with modulated GM images reflecting tissue volume. These images (voxel size: 1 mm^3) were smoothed using a standard Gaussian filter (8 mm FWHM). Analysis was also conducted on images smoothed with a lower Gaussian filter (4 mm FWHM) to increase spatial specificity within MTL structures. We compared GM volume between groups, including age, sex, and TIV as second-level covariates. We report clusters surviving FWE-correction ($p < .05$) at peak-voxel level over $p < .001$, as well as clusters surviving correction for non-stationary smoothness [20] and FWE-correction for cluster size ($p < .05$). The average volumes of these clusters were extracted for all participants using the MarsBaR toolbox [21] (http://marsbar.sourceforge.net/), were residualized for age, sex, and TIV, and entered in bivariate correlation analyses with composite memory scores.

Loane C, *et al. J Neurol Neurosurg Psychiatry* 2019; 0:965–974. doi: 10.1136/jnnp-2018-320168

S5. Resting-state fMRI preprocessing

Resting-state fMRI preprocessing and connectivity analyses were conducted using the CONN toolbox version 17.f (https://www.nitrc.org/projects/conn) [22] for 23/24 patients and 30/33 controls (4 datasets were discarded due to acquisition errors and/or movement). Functional scans were spatially realigned to correct for interscan movement and were slice time-corrected. The structural MRIs and the manually-delineated MTL structures were coregistered to the functional images. Structural images were segmented and normalized along with functional images to the MNI reference brain, followed by motion outlier detection (ART-based scrubbing). Denoising including the anatomical component-based correction method (CompCor) [23] was employed to remove sources of noise in the BOLD time series data, deriving the principal components from the WM and CSF. WM, CSF and the six movement parameters were included as first-level nuisance covariates. A temporal band pass filter (0.01-0.1 Hz) was applied to this residual BOLD signal to remove motion artefacts, physiological and other artefactual effects from the fMRI-signal. Images were smoothed using a 8 mm Gaussian kernel.

Loane C, *et al. J Neurol Neurosurg Psychiatry* 2019; 0:965–974. doi: 10.1136/jnnp-2018-320168

S6. Resting-state functional connectivity (MVPA)

MVPA assesses the multivariate pattern of pairwise connections between all voxels across the whole brain by performing a principal component analysis (PCA) separately for each voxel that characterizes its functional connectivity with the rest of the brain. In the first PCA step, separately for each subject, a default number of 64 PCA components were retained while characterizing each subject's voxel-to-voxel correlation structure. The resulting component scores were then stored as first-level voxel-to-voxel covariance matrices for each subject. In the second PCA step, separately for each voxel and jointly across subjects, the 5 strongest components were retained from a principal component decomposition of the between-subjects variability in seed-to-voxel connectivity maps between this voxel and the rest of the brain, in keeping with a conventionally used conservative 1:10 ratio between the number of components extracted and the number of subjects (n = 53). Subsequently, second-level analyses were performed to test for group differences in whole-brain connectivity by means of an F-test across all MVPA components, comparing for each voxel the component scores across the two groups (covariates: age, sex). The results for each voxel thus reflected between-group differences in functional connectivity between this voxel and the rest of the brain.

Loane C, *et al. J Neurol Neurosurg Psychiatry* 2019; 0:965–974. doi: 10.1136/jnnp-2018-320168

S7. Statistical Analysis

Non-imaging statistical analyses were performed using SPSS (v.25.0, SPSS Inc). Variance homogeneity was assessed using Levene's test, and normal distribution using the Shapiro-Wilk test. Parametric (Student t-test; Welch t-test used when the assumption of homogeneity of variances was violated) and non-parametric tests (when the assumption of normal distribution was not met in a group: Mann-Whitney U for controls vs. patients, and Kruskal-Wallis H for patients with bilateral vs. unilateral vs. no hippocampal atrophy) were used appropriately. To compare neuropsychological test performance between groups, age-scaled, standardized scores were used for independent samples comparisons. The relationship of composite memory scores with GM volumes and functional connectivity measures was investigated with bivariate correlation analyses, using Pearson r and Spearman's ρ (when variables were not normally distributed). Correction for multiple testing for non-imaging data was applied using the Holm-Bonferroni sequential correction method [24].

Loane C, *et al. J Neurol Neurosurg Psychiatry* 2019; 0:965–974. doi: 10.1136/jnnp-2018-320168

Supplementary Table 2: Patients' clinical details

Clinical Variables		Patients (n)	M	IQR
Acute clinical T2-weighted MRI abnormalities	HPC high T2 signal	19/23		
	HPC enlargement	8/23		
	abnormal diffusion	9/17 (facilitated)		
	extra-HPC MTL abnormalities	5/24 (ERC, AMG)		
	extra-MTL abnormalities	1/24 (high T2 signal in caudate nucleus)<br>4/24 (mild microangiopathic changes)		
Seizures	Seizure-free for over a year at time of research study	17/24		
	FBDS (acute)	4/19		
	GTCS	9/19		
	SPS	1/19		
	CPS	13/19		
	MCS	4/19		
	PCS	1/19		
Autoantibodies	LGI1 only	14/24		
	LGI1 + CASPR2	4/24		
	VGKCC	6/24		
	VGKCC-Ab titre at initial clinical presentation (pmol/L)		1353	1452
	acute MRI - antibody testing delay (days)		21	940
Immunosuppressive therapy	Oral steroids	22/24		
	PLEX	9/24		
	IVIG	18/24		
	symptom onset - immunosuppressive treatment onset delay (months)		3	6

AMG: amygdala; CASPR2: anti-contactin-associated protein-like 2; CPS: Complex Partial Seizures; ERC: entorhinal cortex; FBDS: Faciobrachial dystonic seizures; GTCS: generalised tonic-clonic seizures; HPC: hippocampus; IQR: Interquartile Range; IVIG: intravenous immunoglobulin; LGI1: anti-leucine-rich glioma-inactivated1; M: Median; MCS: myoclonic seizures; MTL: medial temporal lobe; PLEX: plasma exchange; SPS: Simple Partial Seizures; VGKCC: anti-voltage-gated potassium channel complex (the 6 patients that had positive VGKCC antibody titres presented prior to the availability of LGI1 or CASPR2 antibody assays).

S8. VGKCC patients

The 6/24 patients that had not been tested for LGI1 or CASPR2 antibodies did not differ from the other 18/24 patients in their acute clinical T2-weighted MRIs (signal intensity, volume, diffusion of left and right hippocampus: all Us, 51 ≥ U ≥ 22; all ps, p-corr > 0.999; occurrence of extra-HPC abnormalities: χ^2 = 0.641, p = 0.423) or post-acutely in any of the neuropsychological tests administered (all Us, 54 ≥ U ≥ 14; all ps, p-corr ≥ 0.231), in their MTL and subcortical volumes (all Us, 53 ≥ U ≥ 24; all ps, p-corr ≥ 0.999), in the mean GM volumes of the clusters identified by VBM (residualized for age, sex, and TIV; all Us, 49 ≥ U ≥ 36; all ps, p-corr > 0.999), or in the mean FC values of the clusters and ROIs identified in the FC analyses (residualized for age, sex, and seed ROI volume; all Us, 48 ≥ U ≥ 32; all ps, p-corr > 0.999).

Loane C, *et al. J Neurol Neurosurg Psychiatry* 2019; 0:965–974. doi: 10.1136/jnnp-2018-320168

S9. Patients with ongoing seizures vs seizure-free patients

Seizure history was recorded and the patient group was dichotomized into those with ongoing seizures (< 1 year since last seizure) and those that had remained seizure-free for over 1 year before participation. The group of 7 patients with ongoing seizures did not differ from the 17 patients that had been seizure-free for over a year in their acute clinical T2-weighted MRIs (signal intensity, volume, diffusion of left and right hippocampus: all Us, $51 \geq U \geq 21$; all ps, p-corr > 0.999; occurrence of extra-HPC abnormalities: $\chi^2 = 0.123$, p = 0.726) or post-acutely in any of the neuropsychological tests administered (all Us, $59 \geq U \geq 21$; all ps, p-corr ≥ 0.561), in their MTL and subcortical volumes (all Us, $58 \geq U \geq 31$; all ps, p-corr ≥ 0.999), in the mean GM volumes of the clusters identified by VBM (residualized for age, sex, and TIV; all Us, $55 \geq U \geq 39$; all ps, p-corr > 0.999), or in the mean FC values of the clusters or ROIs identified in the FC analyses (residualized for age, sex, and seed ROI volume; all Us, $49 \geq U \geq 35$; all ps, p-corr > 0.999).

Loane C, *et al. J Neurol Neurosurg Psychiatry* 2019; 0:965–974. doi: 10.1136/jnnp-2018-320168

S10. Patients with faciobrachial dystonic seizures

The 4/24 patients that had presented with faciobrachial dystonic seizures in the acute phase of the disease did not differ from the other 20/24 patients in their acute clinical T2-weighted MRIs (signal intensity, volume, diffusion of left and right hippocampus: all Us, 34 ≥ U ≥ 19; all ps, p-corr > 0.999; occurrence of extra-HPC abnormalities: $\chi^2 = 2.273$, p = 0.132) or post-acutely in any of the neuropsychological tests administered (all Us, 38 ≥ U ≥ 17; all ps, p-corr > 0.999), in their MTL and subcortical volumes (all Us, 40 ≥ U ≥ 13; all ps, p-corr ≥ 0.925), in the mean GM volumes of the clusters identified by VBM (residualized for age, sex, and TIV; all Us, 40 ≥ U ≥ 38; all ps, p-corr > 0.999), or in the mean FC values of the clusters or ROIs identified in the FC analyses (residualized for age, sex, and seed ROI volume; all Us, 34 ≥ U ≥ 20; all ps, p-corr > 0.999).

Loane C, *et al. J Neurol Neurosurg Psychiatry* 2019; 0:965–974. doi: 10.1136/jnnp-2018-320168

Supplementary Table 3: Correlations of memory scores with hippocampal and thalamic volumes (volumetry)

**Structure**	**Correlations with Memory Composite Scores**					
	**Visual Recall**		**Verbal Recall**		**Verbal Recognition**	
	**r**	**p**	**r**	**p**	**r**	**p**
R HPC head	0.139	0.527	-0.072	0.737	-0.183	0.392
L HPC head	0.157	0.474	0.216	0.310	0.048	0.822
R HPC body	-0.065	0.767	0.079	0.712	0.186	0.385
R Thalamus	0.078	0.724	0.262	0.217	0.087	0.685
L Thalamus	0.047	0.832	0.245	0.249	0.015	0.943

Volumes in which patients showed significant volume reduction were entered in a series of bivariate correlation analyses with memory composite scores (averaged age-scaled standardized scores across subtests in which patients showed impaired performance at group level as compared with controls) for visual recall (ROCFT Immediate and Delayed Recall), verbal recall (WMS-III Logical Memory I and II, Word List I and II, D&P People), and verbal recognition (WMS-III Word List Recognition, RMT Words, D&P Names) across patients; r: Pearson correlation coefficient; p: significance values are presented at uncorrected levels for display purposes. All volumes were TIV-corrected and expressed as z-scores, based on the mean and SD of patients' individually age-matched controls (+/- 10 years of age).

Loane C, *et al. J Neurol Neurosurg Psychiatry* 2019; 0:965–974. doi: 10.1136/jnnp-2018-320168

Supplementary Table 4: Correlations of memory scores with GM volumes (VBM clusters)

Smoothing (FWHM)	FWE-correction level	**Structure**	**Correlations with Memory Composite Scores**					
			**Visual Recall**		**Verbal Recall**		**Verbal Recognition**	
			**r**	**p**	**r**	**p**	**r**	**p**
4mm	peak	L HPC head	0.243	0.264	0.448	0.028	0.211	0.323
		R HPC body	0.051	0.816	0.180	0.401	0.020	0.926
		R HPC head	0.083	0.705	0.171	0.425	0.207	0.332
	cluster size	L HPC head/body	0.188	0.389	0.427	0.038	0.154	0.472
		R HPC head/body	0.028	0.899	0.122	0.569	0.018	0.935
		R Thal	0.286	0.186	0.370	0.075	0.273	0.197
		L/R PCC/PrCu	-0.117	0.594	0.073	0.736	-0.057	0.790
8 mm	peak	R HPC head/body	0.059	0.789	0.173	0.419	0.064	0.767
		L HPC head/body	0.227	0.298	0.438	0.032	0.161	0.454
		R MD Thal	0.250	0.250	0.360	0.084	0.262	0.217
	cluster size	L/R HPC/Thal	0.213	0.330	0.369	0.076	0.145	0.498
		L/R PCC/PrCu	-0.116	0.598	0.089	0.678	-0.084	0.696

The average GM volume from each VBM cluster (in which patients showed significant GM volume reduction) was extracted from each participant, residualised against age, sex, and TIV, and entered in a series of bivariate correlation analyses with memory composite scores (averaged age-scaled standardized scores across subtests in which patients showed impaired performance at group level as compared with controls) for visual recall (ROCFT Immediate and Delayed Recall), verbal recall (WMS-III Logical Memory I and II, Word List I and II, D&P People), and verbal recognition (WMS-III Word List Recognition, RMT Words, D&P Names) across patients. Left hippocampal volumes correlated at uncorrected levels with the verbal recall memory composite scores across patients; r: Pearson correlation coefficient; p: significance values of the bivariate correlations conducted are presented at uncorrected levels; HPC: Hippocampus; L,R: Left, Right; PCC: Posterior Cingulate Cortex; PrCu: Precuneus; MD: Mediodorsal; Thal: Thalamus.

Loane C, *et al. J Neurol Neurosurg Psychiatry* 2019; 0:965–974. doi: 10.1136/jnnp-2018-320168

Supplementary Table 5: Multiple step-wise linear regression analyses

In an exploratory fashion, we assessed the proportion of the variance of patients' verbal recall, verbal recognition, and visual recall memory composite scores that was explained by the patient group's structural and functional abnormalities. We thus entered measures of these abnormalities in a series of multiple step-wise linear regression analyses as independent variables (default alpha level of 0.05 for entry to model and 0.1 for removal). Independent variables were measures of patients' reduced FC between the right hippocampus and the medial prefrontal cortex, the posteromedial cortex, the left hippocampus, the right parahippocampal cortex, and the left temporoparietal cortex, as well as between the right parahippocampal cortex and the left perirhinal cortex; they also included measures of volume reduction in the right, left hippocampus, right and left thalamus, and the posteromedial cortex. In order to examine all different measures of volume reduction in the aforementioned regions (volumes segmented in native space, GM volumes of VBM clusters disclosed at different levels of smoothing and FWE-correction), and in order to minimize multicollinearity, 5 different analyses were conducted for each of those 3 scores, including measures of functional abnormalities, along with measures of volume reduction disclosed by: i) either volumetric measures (table 3); ii) or VBM clusters (cluster-size FWE correction, smoothing at 4 mm FWHM; table 4); iii) or VBM clusters (cluster-size FWE-correction, smoothing at 8 mm FWHM; table 4); iv) or VBM clusters (peak-level FWE-correction, smoothing at 4 mm FWHM; table 4); v) or VBM clusters (peak-level FWE-correction, smoothing at 8 mm FWHM; table 4). All 5 analyses for each of those 3 memory scores showed that patients' reduced hippocampal FC explained the variance of their memory scores.

Loane C, *et al. J Neurol Neurosurg Psychiatry* 2019; 0:965–974. doi: 10.1136/jnnp-2018-320168

Memory Composite Score (Dependent Variable)		Model 1	Model 2
Verbal Recall (z)		R-L HPC FC (z-res)	R-L HPC FC (z-res),<br>R HPC – R PHC FC (z-res)
	R^2	0.214	0.356
	F	5.71	5.52
	p	0.026	0.012
Verbal Recognition (z)		R-L HPC FC (z-res)	-
	R^2	0.466	
	F	18.3	
	p	< 0.0005	
Visual recall (z)		R HPC – Posteromedial Cortex FC (z-res)	-
	R^2	0.299	
	F	8.53	
	p	0.008	

z: age-scaled, standardized scores in memory tests in which patients showed impaired performance at group level as compared with healthy controls; z-res: measures of functional abnormalities (reduced FC between the R HPC and other regions) were residualized across participants against age, sex, and seed ROI volume; FC: functional connectivity; R: right; L: left; HPC: hippocampus; PHC: parahippocampal cortex; -: the step-wise regression was terminated in a single step.

References

1 Nelson HE, Willison J. National Adult Reading Test (NART): Test manual. 1991.

2 Bozeat S, Lambon Ralph MA, Patterson K, *et al.* Non-verbal semantic impairment in semantic dementia. *Neuropsychologia* 2000;**38**:1207–15. doi:10.1016/S0028-3932(00)00034-8

3 Mckenna P, Warrington EK. Testing for nominal dysphasia. *J Neurol Neurosurgery, Psychiatry* 1980;**43**:781–8. doi:10.1136/jnnp.43.9.781

4 Wechsler D. *WASI -II: Wechsler abbreviated scale of intelligence - second edition*. Psychological Corporation 2011.

5 Wechsler D. *Wechsler Memory Scale*. Third. San Antonio, TX: : The Psychological Corporation 1997.

6 Delis DCD, Kaplan E, Kramer JH, *et al. Delis-Kaplan executive function system (D-KEFS)*. Psychological Corporation 2001.

7 Rey A. Manuel du test de copie d'une figure complexe de A. Rey. *Paris Les Ed du Cent Psychol Appliquée* 1959.

8 Warrington EK, James M. *The Visual Object and Space Perception Battery*. 1991.

9 Warrington E. *The Recognition Memory Test*. Windsor, UK: : NFER-Nelson 1984.

10 Warrington EK. *The Camden Memory Tests*. Psychology Press 1996.

11 Baddeley A, Emslie H, Nimmo-Smith I. *Doors and people: a test of visual and verbal recall and recognition*. Bury St. Edmunds, England: : Thames Valley Test Co. 1994.

12 Zigmond AS, Snaith RP. The Hospital Anxiety and Depression Scale. *Acta Psychiatr Scand* 1983;**67**:361–70. doi:10.1111/j.1600-0447.1983.tb09716.x

13 Yushkevich PA, Piven J, Hazlett HC, *et al.* User-guided 3D active contour segmentation of anatomical structures: Significantly improved efficiency and reliability. *Neuroimage* 2006;**31**:1116–28. doi:10.1016/j.neuroimage.2006.01.015

14 Insausti R, Juottonen K, Soininen H, *et al.* MR volumetric analysis of the human entorhinal, perirhinal, and temporopolar cortices. *Am J Neuroradiol* 1998;**19**:659–71.

15 Pruessner JC, Köhler S, Crane J, *et al.* Volumetry of temporopolar, perirhinal, entorhinal and parahippocampal cortex from high-resolution MR images: considering the variability of the collateral sulcus. *Cereb Cortex* 2002;**12**:1342–53. doi:10.1093/cercor/12.12.1342

16 Olsen RK, Palombo DJ, Rabin JS, *et al.* Volumetric analysis of medial temporal lobe subregions in developmental amnesia using high-resolution magnetic resonance imaging. *Hippocampus* 2013;**23**:855–60. doi:10.1002/hipo.22153

17 Ashburner J, Friston KJ. Unified segmentation. *Neuroimage* 2005;**26**:839–51. doi:10.1016/j.neuroimage.2005.02.018

18 Ashburner J, Friston KJ. Voxel-Based Morphometry—the methods. *Neuroimage* 2000;**11**:805–21. doi:10.1006/nimg.2000.0582

19 Ashburner J. A fast diffeomorphic image registration algorithm. *Neuroimage* 2007;**38**:95–113.

20 Hayasaka S, Phan KLL, Liberzon I, *et al.* Nonstationary cluster-size inference with random field and permutation methods. *Neuroimage* 2004;**22**:676–87. doi:10.1016/j.neuroimage.2004.01.041

21 Brett M, Anton J-L, Valabregue R, *et al.* Region of interest analysis using an SPM toolbox [abstract]. In: *8th International Conference on Functional Mapping of the Human Brain*. 2002.

22 Whitfield-Gabrieli S, Nieto-Castanon A. Conn : A functional connectivity toolbox for correlated and anticorrelated brain networks. *Brain Connect* 2012;**2**:125–41. doi:10.1089/brain.2012.0073

23 Behzadi Y, Restom K, Liau J, *et al.* A component based noise correction method (CompCor) for BOLD and perfusion based fMRI. *Neuroimage* 2007;**37**:90–101. doi:10.1016/J.NEUROIMAGE.2007.04.042

24 Holm S. A simple sequentially rejective multiple test procedure. *Scand J Stat* 1979;**6**:65–70. doi:10.2307/4615733
